# Quantitative Analysis of the Impact of Region of Interest Information on Deep Learning Algorithms for Thyroid Ultrasound Imaging

**DOI:** 10.1109/OJEMB.2026.3667415

**Published:** 2026-02-23

**Authors:** Hyunju Lee, Jin Young Kwak, Eunjung Lee

**Affiliations:** Department of Radiology, Severance Hospital, College of Medicine, Research Institute of Radiological ScienceYonsei University 26721 Seoul 03722 South Korea; School of Mathematics and ComputingYonsei University 26721 Seoul 03722 South Korea

**Keywords:** Deep learning, mosaic augmentation, region of interest (ROI), thyroid ultrasonography, YOLOv2

## Abstract

*Goal:* To quantitatively assess the impact of incorporating radiologist-defined Region of Interest (ROI) information in training deep learning models for thyroid ultrasound image classification and lesion localization. Methods: We compared a conventional convolutional neural network (CNN) trained without ROI information, interpreted through Grad-CAM for attention visualization, to Faster R-CNN and YOLOv2 models trained with radiologist-validated ROI masks. We also introduced an adapted mosaic-based composite input, derived from mosaic augmentation but implemented as fixed 1 2 and 2 2 layouts, to improve class balance and spatial diversity in training. Results: Models trained with ROI guidance achieved higher performance in both localization and classification compared to those trained without ROI. The average classification accuracy increased from about 80 in the baseline CNN to around 85 in ROI-guided models that shows an improvement of approximately 5 percentage points. The mean intersection over union between detected and radiologist-defined ROIs increased from approximately 33 to over 70. The adapted mosaic input further stabilized performance across epochs and improved sensitivity while maintaining comparable specificity. Conclusions: Incorporating radiologist-defined ROI information and structured mosaic inputs significantly improves both diagnostic accuracy and localization precision. These results demonstrate that integrating ROI-guided learning with context-preserving composite inputs provides a reproducible framework for developing reliable AI systems in thyroid ultrasonography.

## I. Introduction

Thyroid nodules are extremely common, leading to a continuous rise in the number of individuals seeking medical attention for them. These nodules are distinct anomalies within the thyroid gland, separate from the thyroid parenchyma. While the majority of diagnosed nodules are benign, a minority (approximately 10 to 15) are identified as malignant [1], and this malignancy rate is on the rise. Fine-needle aspiration (FNA), acknowledged globally as the gold standard for diagnosis, is employed to distinguish between benign and malignant thyroid diseases, albeit being an invasive procedure. The advancement of ultrasonography (US) technology has made US the primary diagnostic tool for thyroid nodules [2]. This non-invasive and cheap approach has become the initial step in thyroid testing, resulting in the accumulation of a substantial volume of US image data over time.

As computers have advanced, rapid processing of large datasets has become possible. Consequently, Convolutional Neural Networks (CNNs) have gained prominence, classifying benign and malignant nodules using images alone. However, CNNs inner workings have remained obscure that leave users uncertain about their classification rationale. Researchers thus moved beyond relying solely on large training datasets to enable CNNs to autonomously identify and categorize features. Their goal further includes understanding the factors behind these classifications. Ultimately, many have aimed to develop interpretable AI. Prominent methods for explainable AI in image classification include Gradient-Class Activation Map (Grad-CAM) and Local Interpretable Model-Agnostic Explanation (LIME). Grad-CAM relies on activation-based techniques that use weighted combinations of activations from each convolutional layer [3]. On the other hand, LIME adopts a perturbation-based approach to ascertain importance by altering predicted values based on minor alterations (perturbations) in input data [4]. Employing these methods, one can discern which elements of the input image are deemed significant by the CNN during the classification process. However, despite numerous efforts to apply CNNs and object detection networks to medical ultrasonography, prior studies have seldom investigated how explicitly incorporating radiologist-defined ROI information affects both classification and localization which leaves the quantitative impact of ROI supervision largely unaddressed.

In some regards, Grad-CAM and LIME bear similarities to techniques for locating regions of interest (ROIs). Classical methods have enabled segmentation or detection of ROIs in images, and as research on networks that autonomously learn features from images has progressed, increasingly precise localization models have appeared. Noticeable examples include the U-Net series, the end-to-end fully convolutional network [5]. These models provide ROI information alongside the input, enhancing localization accuracy. They can either automatically identify and extract features from ROIs, or classify the ROI or entire image using CNN training. More recently, simultaneous ROI detection and class classification have become possible through methods such as R-CNN, Fast R-CNN, Faster R-CNN, and YOLO series. Originally developed for diverse, multi-label datasets, these techniques have since been adapted to medical images using transfer learning.

Meanwhile, object detection research has also explored models that utilize multiple images as input. These approaches are generally aimed at enhancing detection performance by leveraging temporal information or integrating multiple views. Notable areas of study include video object detection, multi-view object detection, and image patch merging. Video object detection typically involves stacking sequential frames to provide temporal context as input to the network [6]. Multi-view object detection uses images from different viewpoints to perform object detection in 3D space or from different angles [7]. When the object is small, techniques such as merging patches from multiple images into a single input or duplicating small objects within the same image as part of data augmentation are commonly used [8].

Advanced data augmentation methods, including CutMix [9], [10], [11] and Mosaic Augmentation [12], which combine multiple images for richer and more diverse training inputs, have been widely adopted to improve detection model performance and generalization. In this study, we adapted the mosaic concept to construct a newly suggested composite input structure ($1 \times 2$ and $2 \times 2$ layouts) for thyroid US. Unlike the original on-the-fly mosaic augmentation, our approach predefines spatially arranged inputs that combine benign and malignant nodules within a single image, serving as a controlled multi-context training structure rather than a random augmentation.

**Fig. 1.**
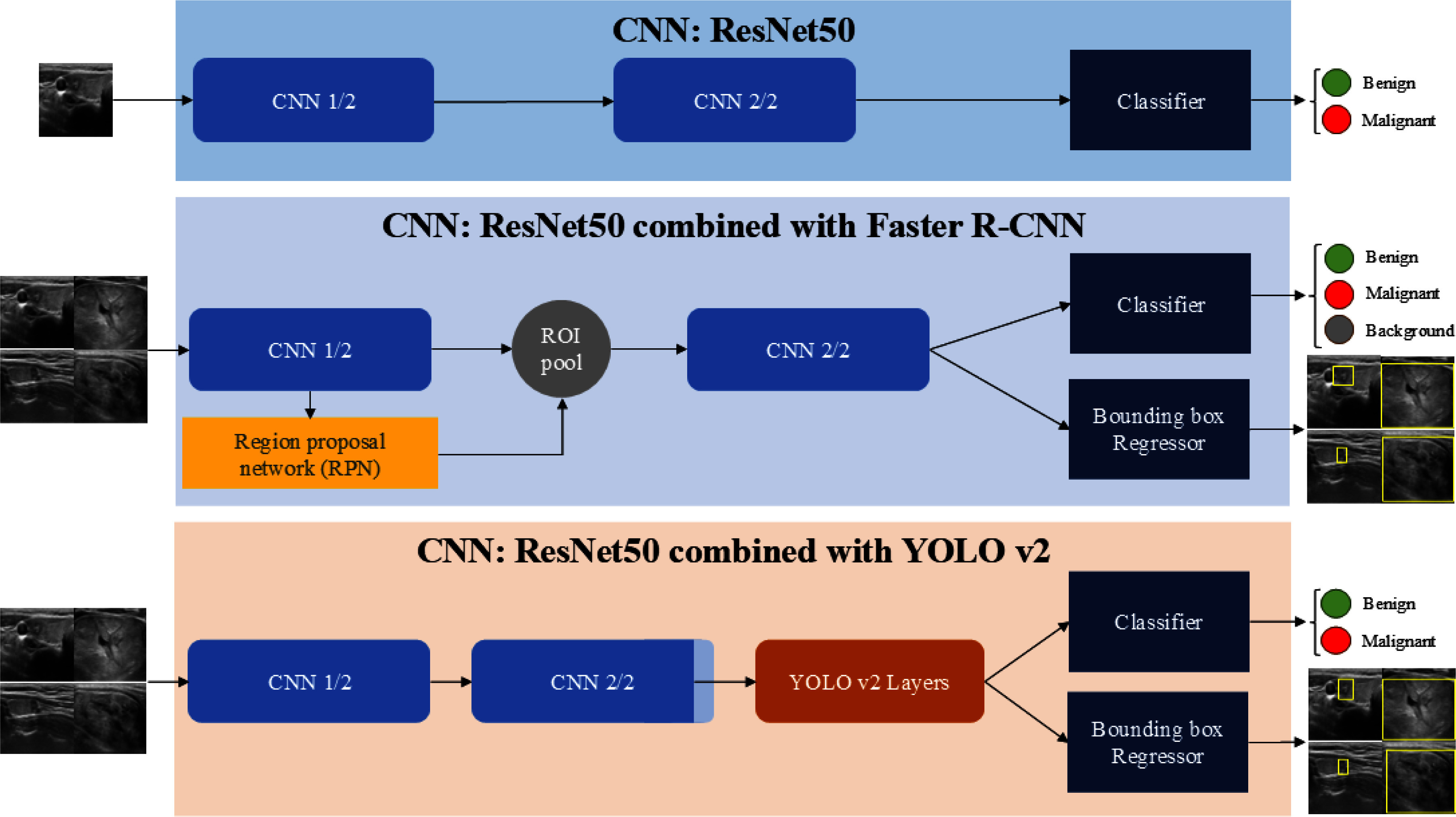
Architectures of three models: CNN, faster R-CNN, and YOLOv2.

This study aims to analyze and quantify the impact of ROI information on CNN thyroid ultrasound image classification and detection. To this end, we conduct comparisons between standard CNNs and detection models (Faster R-CNN and YOLOv2) trained with radiologist-curated ROI masks. Additionally, we introduce and evaluate novel input data structures based on concatenation and augmentation strategies as in Fig. 1. Detailed background and methodological descriptions, including information on deep learning interpretability, object detection architectures, and data augmentation techniques, are provided in the Supplementary Materials.

The present work was designed to address a key research question: whether the incorporation of radiologist-defined ROI information enhances the diagnostic accuracy and localization capability of CNN-based thyroid nodule classifiers, and under what conditions this improvement becomes most significant. Our initial hypothesis was that explicitly training deep learning models with radiologist-annotated ROIs would improve both classification and localization performance compared to CNNs trained without such information. However, we also hypothesized that ROI information alone might be insufficient unless supported by context-preserving composite input structures that capture broader spatial relationships. The main contributions and findings of this study are summarized as follows: (1) We quantitatively present that ROI-guided models outperform conventional CNNs in both localization accuracy and diagnostic consistency. (2) We show that ROI information alone does not guarantee improved performance, whereas combining ROI guidance with mosaic-based input structures enhances both classification accuracy (by 45) and stability across epochs. (3) We introduce a new mosaic input strategy that effectively integrates multiple nodule regions into a single input, enriching contextual representation. (4) We provide a unified, reproducible framework for evaluating the impact of ROI information on both detection and classification performance in medical imaging.

## II. Materials and Methods

### A. Experimental Data

Our research uses thyroid US images from Severance Hospital, Yonsei University Health System (approved by the institutional review board (IRB) of Severance Hospital, Seoul, South Korea, with a waiver of informed consent). Images were collected from patients who underwent fine-needle aspiration (FNA) or surgery between June 2012 and March 2019. Patients aged 18 and older were included. Thyroid nodules with a maximum diameter over 1 cm and confirmed benign or malignant diagnoses by cytology or pathology were considered. The dataset contains 13967 US images, split into 13333 training and 634 test images. The dataset has been used since February 18, 2025. The training set includes 6400 benign and 6933 malignant cases; the test set, 95 benign and 539 malignant. Mask images marking the ROI for each original image were obtained under radiologist supervision. Original images were captured from the US devices monitor; thus, preprocessing was applied to remove non-US elements like letters, symbols, and the gray scale bar to ensure data integrity.

### B. Various Input Structures

We proposed new input data structures for Faster R-CNN [13] and YOLOv2 [14] to improve detection and classification performance. While the base CNN and detection models utilized the same training dataset, Faster R-CNN and YOLOv2 required specific input formats to facilitate multi-label learning in MATLAB. To address this, benign and malignant nodule images were merged to create composite images:

• Class balancing was achieved via oversampling.
• Benign and malignant images were concatenated side-by-side; larger composites (e.g., two benign and two malignant images arranged in a square) were also generated.
• Rotational arrangements were created to ensure all regions and label positions were learned.

To ensure reproducible ROIlabel alignment within the composite input structures, each sub-image preserved its original diagnostic label (benign or malignant) and corresponding ROI coordinates. During the construction of the 2 2 mosaic inputs, two benign and two malignant nodules were systematically arranged at fixed spatial positionsfor instance, benign nodules were placed at (11) and (22), while malignant nodules occupied (12) and (21). The ROI bounding boxes from each sub-image were geometrically transformed to the new global coordinate system by adjusting their upper-left coordinates and preserving their original width and height. All possible rotational arrangements (90, 180, and 270) of these four regions were also generated to enrich spatial diversity.

As a result, each composite image contained four consistently aligned bounding boxes with accurate label correspondence, ensuring that every nodules diagnostic label and ROI position remained traceable and reproducible across the entire dataset.

**Fig. 2.**
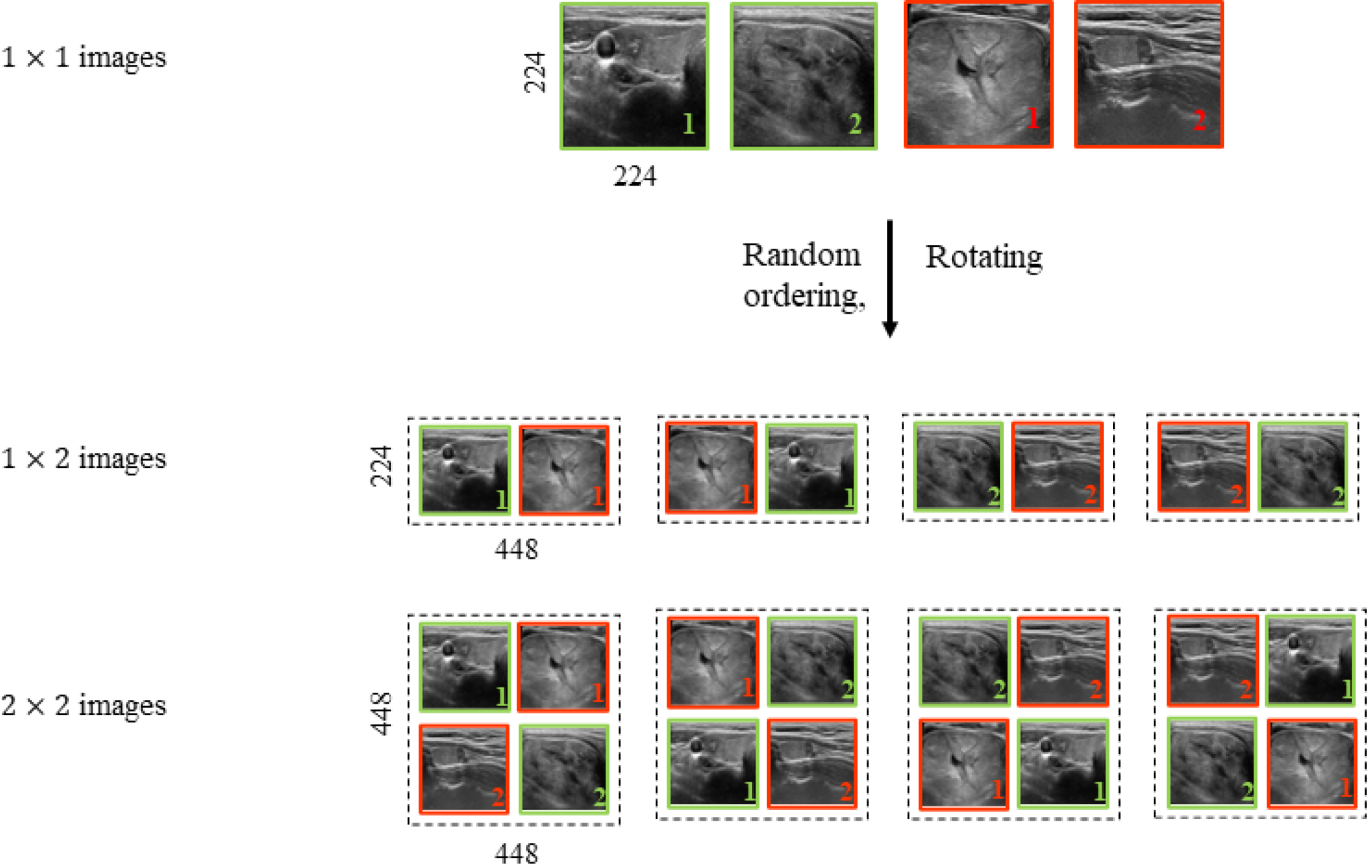
Input data structures for CNN with faster R-CNN and YOLOv2.

The process for constructing these composite input structures is illustrated in Fig. 2. As shown, $1 \times 2$ and $2 \times 2$ layouts enable the detection models to identify and learn from multiple nodule regions within a single image. These modifications enabled robust training of detection models and improved performance, as discussed in the Results section.

### C. Experimental Settings

ResNet-50 [15], a widely used convolutional neural network, was selected as the backbone model for all experiments based on its strong validation performance with our thyroid ultrasonography (US) dataset. For classification, ResNet-50 was adapted for binary output by replacing its final layers and trained using cross-entropy loss. For object detection, both Faster R-CNN and YOLOv2 utilized ResNet-50 as their backbone, with task-specific modifications including new classification heads and detection modules. All training was performed on an NVIDIA RTX 3090 GPU using MATLAB R2022a. Hyperparameters such as batch size and number of epochs were optimized for each model to balance accuracy and training time.

Training hyperparameters for ResNet-50, Faster R-CNN, and YOLOv2, including batch size, learning rate, momentum, weight decay, and number of epochs, are summarized in Table I. The random seed was not explicitly fixed for each training session. Nevertheless, all models were trained under identical hardware and data loading conditions, and repeated trials confirmed consistent convergence and negligible variance in performance. Instead of using k-fold cross-validation, a fixed training/validation split was defined once from the original dataset and consistently applied across all experiments, ensuring that every model was trained and validated on the same data partitions for a fair comparison.

**TABLE I:** Experimental Settings for Training ResNet-50, Faster R-CNN, and YOLOv2

Option	ResNet-50	ResNet-50	
		Faster R-CNN	YOLOv2
Optimizer	SGDM	SGDM	SGDM
Batch size	16	2	16
Max epoch	40	3	40
Learning rate	0.001		
Momentum	0.9 (default)		
Weight decay	0.0005 (default)		
Augmentation	Random left-right flip and scaling within the range 11.1		

For clarity, the main parameters and their purposes are summarized as follows. The learning rate (1 10^3^) controlled the weight update step size, while momentum (0.9) and weight decay (0.0005) regularized optimization. Mini-batch sizes of 16 (YOLOv2) and 2 (Faster R-CNN) were chosen according to GPU memory constraints. Positive and negative region proposals for Faster R-CNN were defined using$IoU \geq 0.6$ and $IoU \leq 0.3$ thresholds, respectively (used only for labeling training proposals). Three anchor boxes were estimated from the training data to guide region proposals. Detection losses combined cross-entropy for classification and Smooth L1 for bounding box regression, while YOLOv2 additionally used mean squared error for coordinate prediction. Classification performance was evaluated using accuracy, sensitivity, and specificity, averaged over epochs 3140 (mean standard deviation). Localization performance was quantified by Intersection over Union ($IoU$), Intersection over ROI ($IoR$), and Intersection over Detected area ($IoD$).

### D. Detection and Classification Methods

Three models were compared: ResNet-50 trained solely for benign/malignant classification without ROI information, and ResNet-50 combined with Faster R-CNN or YOLOv2, which perform detection and classification using radiologist -annotated ROI masks.

For the detection models, output bounding boxes defined detected regions. Since ResNet-50 is a classification-only network, Grad-CAM was applied to visualize attention regions relevant to classification decisions. These attention heatmaps highlight image areas weighted most heavily during prediction.

Detection models and ResNet-50 were trained on various input image structures: YOLOv2 on $1 \times 1$ and $2 \times 2$ square inputs, Faster R-CNN on $1 \times 2$ and $2 \times 2$ inputs, respecting each models input constraints. Classification results from each detected region were aggregated for final image-level prediction.

A detailed description of model architectures, training setups, Grad-CAM implementation, and classification aggregation methods is provided in the Supplementary Materials.

## III. Results

### A. Metrics

The classification performance was evaluated using accuracy, sensitivity, and specificity, computed as

\begin{equation*}
 Accuracy = \frac{{TP + TN}}{{TP + TN + FP + FN}}, 
\end{equation*}

\begin{equation*}
 Sensitivity = \frac{{TP}}{{TP + FN}},\,Specificity = \frac{{TN}}{{TN + FP}}, 
\end{equation*}

where TP, TN, FP, and FN denote true positives, true negatives, false positives, and false negatives, respectively.

To quantify localization performance, we introduced three region overlap metrics comparing detected regions ($D$) with expert radiologist ROIs ($R$):

• Intersection over Union ($IoU$): $\frac{{R \cap D}}{{R \cup D}} \times 100$
• Intersection over ROI ($IoR$): $\frac{{R \cap D}}{R} \times 100$
• Intersection over Detected Area ($IoD$): $\frac{{R \cap D}}{D} \times 100$

**Fig. 3.**
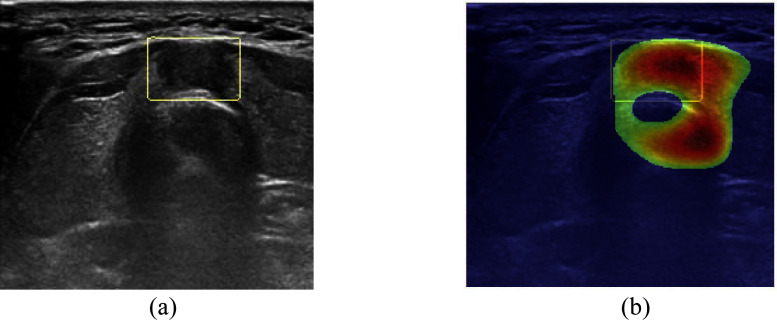
A case of malignant image classified as malignant by ResNet-50. (a) is the original image indicated the ROI and (b) is the top 50 area of heat map by ResNet-50.

In this paper, we have selected $IoU$ as our primary evaluation metric to assess the algorithms capacity for accurate localization. More specifically, it enables us to determine whether the algorithm correctly identifies the appropriate region to make an accurate classification decision. It is worth noting that all three metrics could be applied, but our preference for $IoU$ stems from its aptness for identifying cases in which the CNN might have focused on areas unrelated to the task, even if it arrived at a correct decision. Fig. 3 shows an instance in which the CNN predominantly concentrates on three specific areas, ultimately concluding that it is an image of malignancy, aligning with the gold standard. However, it remains ambiguous whether the CNNs decision to classify the image as malignant is primarily influenced by features from the area where there is an overlap with the radiologist-indicated ROI or if the decision is derived from an incorrect region, as depicted in Fig. 3(b). Evaluating the three metrics with values of $IoU$ 0.3380, $IoR$ 0.8582, and $IoD$ 0.3580, it becomes evident that $IoU$ is the most informative metric, suggesting that the CNN may, at times, consider irrelevant areas during its decision-making process. In addition, for a subset analysis described in the Supplementary Materials, we evaluated how many true-positive (TP) and true-negative (TN) detections achieved an $IoU \geq 30\% $, which was used as a practical threshold to indicate sufficiently accurate localization. This analysis allowed us to assess whether both correctly diagnosed malignant and benign images were based on regions consistent with radiologist-defined ROIs.

### B. Analysis of Algorithm Speed

We evaluated the computational efficiency of the models by measuring their average training time per epoch and testing time per image on the same hardware platform. As summarized in Table II, ResNet-50 demonstrates the fastest training and inference speeds among compared models. Faster R-CNN requires substantially longer training times due to its region proposal network, especially with larger input structures. YOLOv2 offers a favorable balance, providing significantly faster training and testing than Faster R-CNN while maintaining strong performance.

**TABLE II:** Comparison of Speed (h: Hour, m: Minute, s: Second)

		ResNet-50	ResNet-50			
			Faster R-CNN		YOLOv2	
		$1 \times 1$	$1 \times 2$	$2 \times 2$	$1 \times 1$	$2 \times 2$
Train	Average training time per epoch	10m	8h	19h	5m	15m
	Speedup	114x	2.4x	1x	228x	76x
Test	Average testing time per image	0.0036s	0.1127s	0.2243s	0.0039s	0.2220s
	Speedup	62.3x	2x	1x	57.5x	1x

### C. Detection and Classification Results

**Fig. 4.**
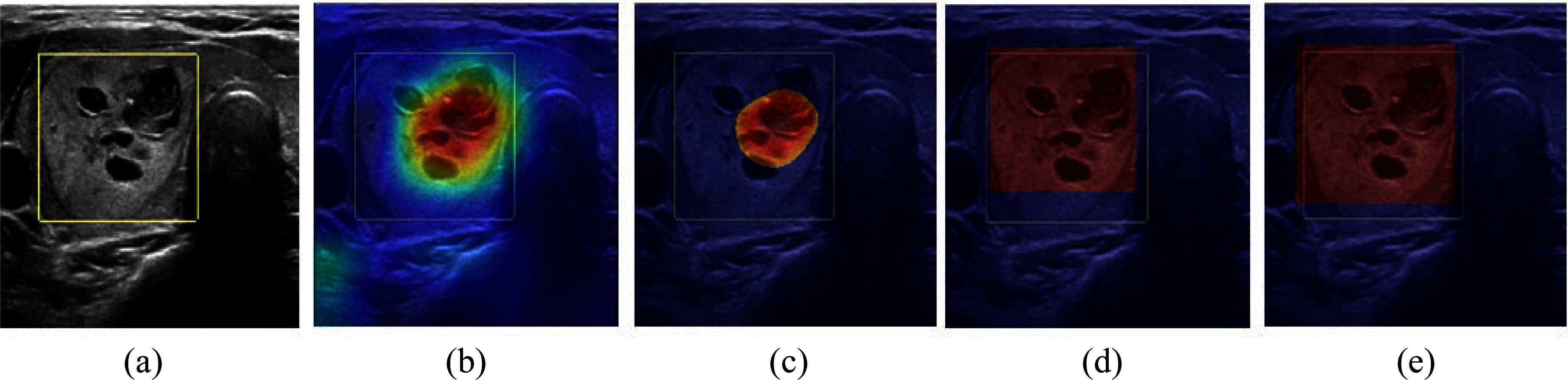
Comparison of detected regions by three models. (a) is the original image with ROI, (b) is the heat-map using Grad-CAM for ResNet-50, (c) is top 30 of the heat-map of ResNet-50, (d) is the bounding box by Faster R-CNN, and (e) is the bounding box by YOLOv2.

The Grad-CAM score map quantifies the level of influence a specific layer has on the classification result, represented as a differential value. In Fig. 4(b), one can observe the score map for an image displayed as a heatmap. We computed three metrics ($IoU$, $IoR$, and $IoD$) for the top 30, 50, and 70 of the score map and the detected area identified by Faster R-CNN and YOLOv2. Table III displays the average percentage of overlapping ratios across all 634 test images. Comparative examination with the results obtained from ResNet-50 in conjunction with Faster R-CNN and YOLOv2 configurations reveals that the original ResNet-50 exhibits reduced overlapping areas. These findings provide evidence that the localization capability of the CNN trained with ROI information surpasses that of its counterpart without ROI data.

**TABLE III:** Comparison of Detection Results for ResNet-50, Faster R-CNN, and YOLOv2

Metric	ResNet-50			ResNet-50	
	Top 30 of score map	Top 50 of score map	Top 70 of score map	Faster R-CNN	YOLOv2
$IoU$	20.23	31.54	38.13	67.44	71.93
$IoR$	22.01	38.89	58.50	81.12	81.33
$IoD$	75.96	68.86	57.98	82.11	88.17

Next, we conduct a comparative examination of the classification performance between ResNet-50 and ResNet-50 equipped with detection models using different input data structures. When the classification CNN is paired with a detection model, one can modify the input structure to enhance overall performance in both localization and classification. In this context, we use the classification result corresponding to the (1, 1) region among the four detected regions in the $2 \times 2$ input structure of Faster R-CNN, as it yielded superior performance compared to the individual classification results from other regions or any combination thereof. As for YOLOv2, the final classification result was determined by aggregating the classification outputs from all detected regions within the $2 \times 2$ input. The outcome of this approach shows that the detection model using a $2 \times 2$ input structure, combined with the CNN, produces higher accuracies than those using fewer images. Consequently, we advocate the adoption of $2 \times 2$ images as the input structure for the detection model.

Furthermore, the classification results displayed in Table IV reveal that ResNet-50, when coupled with Faster R-CNN and YOLOv2, trained with ROI data using $2 \times 2$ images, achieved approximately 4 to 5 higher accuracy compared to ResNet-50 which was exclusively trained on images without ROI information. For ResNet-50 and YOLOv2, classification metrics were averaged over epochs 3140, and the mean standard deviation were reported to reflect performance stability during the convergence stage. Detailed per-class localization results, including the proportion of correctly localized malignant (TP) and benign (TN) detections with $2 \times 2$, are presented in Supplementary Tables S-2 and S-3, providing class-wise quantitative validation of localization accuracy. The detailed training and validation curves, along with confusion matrices for each model and input configuration, are provided in the Supplementary Materials (Figures S-1 and S-2), confirming stable convergence and consistent class-wise prediction patterns.

**TABLE IV:** Comparison of Classification Results for ResNet-50, Faster R-CNN and YOLOv2. Values for ResNet-50 and YOLOv2 Models Represent the Mean Standard Deviation Over Epochs 3140, Where Performance Was stable. This Approach Quantifies the Variability Across Epochs to Ensure Reliability of the Reported Metrics.

Model	ResNet-50	ResNet-50			
		Faster R-CNN		YOLOv2	
Epoch	31-40	3		31-40	
Input structure	$1 \times 1$	$1 \times 2$	$2 \times 2$	$1 \times 1$	$2 \times 2$
Accuracy	0.8038 $ \pm $ 0.0148	0.8375	0.8549	0.7090 $ \pm $ 0.0979	0.8446 $ \pm $ 0.0089
Specificity	0.7726 $ \pm $ 0.0223	0.7684	0.8000	0.8653 $ \pm $ 0.0973	0.8000 $ \pm $ 0.0302
Sensitivity	0.8093 $ \pm $ 0.0165	0.8497	0.8646	0.6814 $ \pm $ 0.1314	0.8525 $ \pm $ 0.0120

ROI Detection Analysis was conducted to evaluate the agreement between model-detected regions and expert-annotated ROIs. While the detailed analysis, including quantitative overlap metrics and comparative evaluations between Grad-CAM attention maps and bounding box detections, is presented in the Supplementary Materials, the main manuscript focuses on overarching detection and classification performance trends. Models trained with expert ROI information demonstrate notably higher localization accuracy and diagnostic reliability.

For a comprehensive examination of ROI detection performance, including detailed metrics and visualization examples, please refer to the Supplementary Materials.

## IV. Discussion

Our study clearly presents that a model trained to recognize ROI is capable of more accurate ROI detection and classification compared to a model lacking ROI training. We substantiated this claim by quantifying classification and detection accuracies for each model. The superior performance of the ROI-trained model in Table IV can be attributed to insights gained from Table III and Fig. 4. Upon a careful examination of the data presented in the second to fourth columns of Table III, it is evident that a deep learning algorithm trained without ROI information is more likely to incorporate irrelevant data into its decision-making process. Consequently, the overall accuracy of models trained with ROI information is higher, as they focus on the pertinent region when making decisions.

To further corroborate this analysis, a comprehensive investigation was undertaken to assess the ROI detection and result classification. Tables IV, S-2, and S-3 show the outstanding performance of Faster R-CNN and YOLOv2 in pinpointing the correct ROIs, consequently leading to more precise classification outcomes. Impressively, these models differ by only about 3-5 in recognizing the critical regions. Nevertheless, upon inspecting the original images in Figure S-4 (a)(c), a notable pattern emerges. This indicates that ResNet-50, which was trained without ROI information, sometimes excels in classification but does not heavily depend on the actual ROIs. Instead, it leans on non-nodule areas to make its decisions. This pattern also manifests in Figure S-4 (c)(d), where ResNet-50, lacking ROI information, consistently misclassifies images, resulting in a decrease in overall accuracy.

A comprehensive analysis of Tables II, III, and IV demonstrates that combining ResNet-50 with Faster R-CNN or YOLOv2 and utilizing $2 \times 2$ input structure yields superior performance in both detection and classification compared to using standalone ResNet-50. Furthermore, within each model, the $2 \times 2$ input structure consistently outperforms other data configurations (e.g., $1 \times 1$ or $1 \times 2$). For instance, Faster R-CNN achieved the highest accuracy, specificity, and sensitivity values with the $2 \times 2$ input structure, while YOLOv2 demonstrated the best accuracy and sensitivity with the $2 \times 2$ input structure, although its specificity was slightly higher with the $1 \times 1$ input structure, as seen in Table IV. These findings show how effective this structure is in capturing diverse ROIs and enhancing the representation of important areas, ultimately improving performance.

While our initial hypothesis assumed that training with ROI information alone would improve diagnostic performance, the results revealed a more nuanced outcome. ROI guidance alone did not consistently yield higher accuracy compared to baseline CNNs which indicates that region-specific supervision, when isolated from spatial context, may not fully leverage its potential. However, when ROI information was integrated with mosaic-based composite inputs, both detection and classification performances improved markedly. This finding highlights that ROI learning benefits most when combined with context-aware input structures that preserve inter-regional relationships within thyroid nodules.

These improvements are clinically meaningful, as more accurate and spatially focused detection of thyroid nodules can reduce false diagnoses and assist radiologists in confirming lesion boundaries with higher confidence. Although the present study was based on a single-institution dataset, the reproducible framework and consistent trends across different architectures suggest strong potential for generalization to multi-center clinical settings.

However, when examining the training and testing time, Faster R-CNN shows significantly lower efficiency. This indicates that integrating YOLOv2 with ResNet-50 could be a more effective approach for developing real-time, high-performance models for medical imaging. Moreover, the improved classification and detection performance observed with the integration of YOLOv2 was also evident for GoogLeNet and Inception-v3, as demonstrated in Tables S-4 and S-5 in the Appendix. Nevertheless, not all CNN architectures perform equally well when combined with YOLOv2. While many CNNs can be integrated with YOLOv2, the resulting performance heavily depends on their feature extraction capabilities and computational efficiency. For instance, shallow networks like LeNet fail to provide the high-level features required for detecting complex objects, whereas extremely deep networks such as ResNet-101 or ResNet-152 are computationally expensive and therefore unsuitable for real-time YOLOv2 applications.

## V. Limitations of the Study

While this study presented that integrating radiologist-defined ROI information and mosaic-based composite structures improves diagnostic accuracy and localization performance, some of the limitations should be acknowledged. First, the prolonged training time required by the Faster R-CNN model led to notable computational constraints. Second, ROI-guided improvements were validated only within a specific set of network architectures (ResNet-50, Faster R-CNN, and YOLOv2), and may not generalize equally to other backbone models or detection frameworks. Finally, all experiments were performed using data from a single institution, and external validation with multi-center datasets will be required to confirm generalizability and clinical robustness. Future work will address these limitations by expanding the dataset scope, exploring adaptive mosaic generation strategies, and validating the proposed framework on additional architectures.

## VI. Conclusion

In this study, we conducted a comprehensive analysis of detection and classification performance, comparing CNN (ResNet-50) with and without ROI recognition (Faster R-CNN and YOLOv2). This allowed us to examine how the presence or absence of ROI information impacted the results. Notably, the classification performance of the model combining ResNet-50 with detection models surpassed that of the conventional ResNet-50 image classifier.

We evaluated three models: ResNet-50, ResNet-50 with Faster R-CNN, and ResNet-50 with YOLOv2. The models identified key regions, revealing differences in detecting malignancies. ResNet-50, lacking specific region training, often correctly identified malignant images but focused on areas distant from the actual nodule. In contrast, Faster R-CNN and YOLOv2 enabled decisions over regions overlapping radiologist-defined ROIs in over 96 of true positive cases, emphasizing integration of radiologist expertise into CNNs via ROIs. Support is found in other deep learning models (see Supplementary Tables S-4 and S-5).

When benchmarked against recent studies from 20242025 (Supplementary Table S-6), our framework achieved comparable or higher localization accuracy and interpretability despite using a smaller, single-institution dataset. Whereas most contemporary approaches such as multi-view YOLO models [16], DETR variants [17], and either multimodal [18] or multi-center frameworks [19] focus on expanding model architecture, our work demonstrates a reproducible and explainable strategy through ROI-guided and mosaic integrated learning.

Through results in Table IV, with insights from Table S-2, S-3, and Figure S-3, we show that greater overlap between regions $D$ and $R$ leads to improved diagnostic accuracy. This study shows that combining ROI information, especially with advanced architectures like Faster R-CNN and YOLOv2, delivers superior medical imaging performance. Incorporating ROI information in training is critically important for detection and classification, and ROI-focused training with augmentation further enhances diagnostic precision.

## Supplementary Materials

Supplementary Materials include detailed descriptions of related works (ResNet-50, Faster R-CNN, YOLOv2), extended experimental data and preprocessing procedures, and descriptions of detection and classification methods. Additional results are also provided, including algorithm speed analysis, ROI detection evaluation, confusion matrices for each model, training-validation curves of loss, accuracy, and RMSE, ablation studies on ROI and mosaic integration, and per-class localization statistics for true-positive and true-negative cases. Furthermore, experiments with alternative CNN backbones (GoogLeNet and Inception-v3) and a comparative literature review summarizing recent 20242025 studies are presented (Table S-6).

Supplementary Materials

## Authors Contributions

All authors contributed to the study conception and design. Material preparation and data analysis were performed by Hyunju Lee and Eunjung Lee. Data collection was performed by Jin Young Kwak. The first draft of the manuscript was written by Hyunju Lee and all authors commented on previous versions of the manuscript. All authors read and approved the final manuscript.

## Ethics Declarations

The institutional review board (IRB) of Severance Hospital, Seoul, South Korea granted approval for this retrospective study with a waiver of the requirement for informed consent.
