# Supplementary material for: Quantitative Analysis of the Impact of Region of Interest Information on Deep Learning Algorithms for Thyroid Ultrasound Imaging
Source: IEEE Open J Eng Med Biol. 2026 Feb 23;7:172–9. doi: 10.1109/OJEMB.2026.3667415 (PMC13175602; doi:10.1109/OJEMB.2026.3667415)
Supplement: Supplementary Materials [file supp1-3667415.doc]

# [[1]](#footnote-2)  Related Works

## ResNet-50

Prior to the emergence of residual networks, prevalent models employed for image classification were characterized by a repeated stacking of convolutional layers. These models grappled with several issues, including challenges in optimization, vanishing gradient dilemmas, and performance degradation. The paper ‘Deep residual learning for image recognition’ [15] introduced an innovative solution in the form of a ‘residual block’, which incorporated skip connections referring to the input of the preceding layer within the subsequent layers. ResNet-50, boasting a deep architecture comprising 50 layers with these residual blocks, successfully mitigated the notorious problems associated with vanishing and exploding gradients that typically plague the training of deep convolutional neural networks (CNNs). As a result, ResNet-50 has gained widespread adoption and acclaim for its exceptional performance in the realm of classifying ultrasonography (US) images [20,21].

## Faster R-CNN

Faster R-CNN [13] comprises two essential modules. The initial module is a deep fully convolutional network responsible for proposing regions of interest, while the second module serves as the detector, leveraging these proposed regions. In its operation, Faster R-CNN harnesses a pre-trained convolutional neural network to extract features from the input image. Subsequently, the regions proposed by the first module are mapped onto the features derived from the initial portion of the CNN. These mapped regions then traverse the remaining layers of the CNN, facilitating the pixel-wise classification of the image into various classes. As a result, this approach yields both the proposed regions and their corresponding labels.

## YOLOv2

YOLOv2, short for 'you-only-look-once' [14], introduces a novel approach to object detection, predicting bounding boxes and class probabilities simultaneously within a single-stage detection network. This design inherently grants YOLOv2 a significant speed advantage over Faster R-CNN, which relies on a convolutional neural network for region proposal. By employing a CNN as its foundation, YOLOv2 extracts features in a manner akin to traditional classification models. Furthermore, the integration of a YOLOv2 layer at the conclusion of the CNN generates the final output, encompassing critical information such as the bounding box's center point, width, and height, along with the bounding box's confidence score and the class probability associated with each bounding box.

# Extended Experimental Data and Preprocessing

## ROI annotation and additional experimental data

Mask images delineating the regions of interest (ROI) for each original US image were acquired. This annotation process was meticulously overseen by radiologists to ensure accuracy. Since the original images were captured from the US imaging device’s monitor during thyroid examinations, they contained non-US elements such as letters, symbols, and grey color scale bars. In the subsequent subsection, we elaborate on the details of this preprocessing procedure.

## Preprocessing

In the case of training a standard CNN, an input image is expected to carry just one label, either 'benign' or 'malignant'. To meet this requirement, we meticulously processed our images, eliminating any non-US elements that could potentially disrupt the CNN training process. The images preprocessed in the following steps are used as input data for CNNs.

1. Remove non-ultrasound overlays and borders surrounding the original images.
2. Normalize the intensity values of the US image.
3. Resize the normalized image to
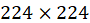


Preparing input images for Faster R-CNN and YOLOv2 involves additional information compared to conventional CNNs. An additional input is a mask (ground-truth) image showing the ROI. Such mask and preprocessed US images are paired and used as input data for Faster R-CNN and YOLOv2.

## Various input structures

The new input data structures, categorized as a form of Mosaic Augmentation, offer several advantages: First, the data distribution is expanded through concatenating multiple images. Through concatenation, the resulting distribution captures a wider variety of diverse distributions. This expansion of the data space enables learning across a broader sample space, effectively mitigating overfitting. Moreover, processing multiple diverse images within a single batch improves the efficiency of Batch Normalization, as the batch inherently contains more varied data. Using concatenated images allows processing multiple images simultaneously, mimicking the effect of a larger batch size. This improves learning stability and speeds up processing, similar to training with a larger batch size. Additionally, objects near the image border can be effectively relocated to a location farther from the new edge through the concatenation process, facilitating more robust learning. Finally, combining images with different labels into a single input allows the model to learn multiple labels simultaneously, addressing class imbalance by reducing the dominance of a specific class. This approach ensures a more balanced representation of label, which further improves model generalization.

## Experimental settings

In MATLAB, a pretrained ResNet-50 (trained on ImageNet) was used, which comprises 177 layers including batch normalization, convolutional blocks, residual skips, and fully connected layers. For binary classification, the last three layers were replaced, and cross-entropy loss was used.

For Faster R-CNN, ResNet-50's last layers were modified to predict three classes (two object types plus background); the feature extraction layer was set to ‘activation_40_relu’. Detection used a combined loss of cross-entropy (classification) and Smooth L1 (bounding box regression).

YOLOv2 also used ResNet-50 as a backbone, with final layers removed. The network used both high-level (‘activation_49_relu’) and low-level (‘activation_47_relu’) feature maps, merging them via a reorganization layer for anchor-based detection. YOLOv2 loss combined mean squared error (bounding box), binary cross-entropy (objectness), and categorical cross-entropy (classes).

Due to Faster R-CNN’s longer training time, the batch size was reduced to 2 and the number of epochs was limited to 3. YOLOv2 used a larger batch 16, trained for 40 epochs; ResNet-50 classification also trained for 40 epochs. The performance metric for YOLOv2 and ResNet-50 was averaged over the last 10 (of 40) epochs for stability, while Faster R-CNN’s final epoch (3) was used as representative due to convergence speed. All data augmentation included random horizontal flip and scaling within a specified range.

## Supplementary convergence analysis

For completeness, additional experiments were performed to visualize the convergence characteristics of the learning process. These results are intended to supplement the main findings by presenting typical training and validation trends of the implemented models.

Here, training sessions were conducted using the same datasets and hyperparameters as in the main experiments, but on currently available computing systems: MATLAB R2022a with an NVIDIA GeForce RTX 2080 GPU for the ResNet-50 (
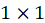
) configuration, and MATLAB R2025b with an NVIDIA GeForce RTX 5090 GPU for the YOLOv2 (
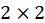
) and Faster R-CNN (
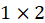
) configurations. Although the hardware and MATLAB versions differed from those used in the original setup, the resulting convergence behaviors and final performance metrics were consistent with those reported in the main text.

Due to computational resource constraints, YOLOv2 (
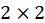
) was trained for 10 epochs, and the Faster R-CNN (
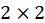
) configuration was replaced with the (
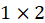
) variant. In MATLAB, YOLOv2 provides both root-mean-square error (RMSE) and loss values for training and validation phases but does not track accuracy during training. In contrast, ResNet-50 and Faster R-CNN support simultaneous visualization of accuracy and loss for both training and validation.

Accordingly, each plot in Figure S-1 displays both training and validation curves for its respective metrics:

(a) ResNet-50 (
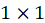
): accuracy and loss,

(b) YOLOv2 (
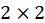
): RMSE and loss,

(c) Faster R-CNN (
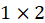
): accuracy and loss.

This supplementary session was performed solely to reproduce the convergence plots shown in Figure S-1 and do not affect or alter the primary experimental results reported in the manuscript.

## Detection and classification methods

For Faster R-CNN and YOLOv2, the output bounding box is used as the detected region. As the original ResNet-50 model only yields classification results, we employed Grad-CAM to visualize the regions deemed significant in its decision-making process. These vital regions can be visually confirmed by applying weights obtained through Grad-CAM to the input image, which is represented as a heatmap. In this context, we are focused on identifying the top percentage of total weight as the detected region.

To train Faster R-CNN and YOLOv2, we evaluate the classification results on input images with various structures. When assessing the detection model's performance on
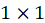
 images, we rely on YOLOv2. In this specific scenario, the detector is trained to recognize a single label, "nodule," encompassing both benign and malignant cases. Since each image carries only one label, MATLAB suffices for training the detection model. Later, we enhance the model by appending a classifier for distinguishing between benign and malignant nodules. However, it's worth noting that adding a classifier directly to the end of Faster R-CNN model is challenging, so the experimentation with
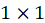
 images was exclusively carried out with YOLOv2. For
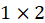
 images, we exclusively train Faster R-CNN, as YOLOv2 mandates square-shaped input structures. Subsequently, both models are trained using
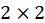
 images.

To evaluate detection models trained on combined images created by repeatedly pasting one test image to match the input data size, we generate test images with two square regions, each with a side length of 224 for
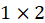
 images, and four square regions for
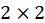
 images. The final result is determined based on the classification outcomes for each region and a combination of these results.

# Results

## Detailed analysis of algorithm speed

To assess the model's efficiency, we evaluated the training time, ensuring it was measured consistently on the same machine. In Table II, we present a comparison of the execution speed between ResNet-50 and ResNet-50 equipped with Faster R-CNN and YOLOv2. ResNet-50 takes approximately 10 minutes to complete training for a single epoch. On the other hand, Faster R-CNN, which involves a convolutional network for region proposals, requires significantly more time, necessitating 8 hours and 19 hours per epoch for
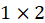
 and
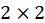
 input image structures, respectively. YOLOv2, which directly predicts bounding boxes without the region proposal network used by Faster R-CNN, offers a relatively shorter training period of 5 minutes and 15 minutes per epoch for
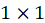
 and
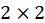
 input image structures, respectively. In the case of YOLOv2 trained on
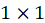
 images, the initial training focused on nodule detection without class distinctions, taking 5 minutes per epoch. Subsequently, a classifier was integrated into the pre-trained network, leading to image classification into two classes, a process that also took approximately 10 minutes per epoch, aligning closely with training time of ResNet-50.

Table II also includes information on the processing time for testing a single image. Both Faster R-CNN and YOLOv2, utilizing the larger
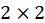
 input structure, exhibit testing times of about 0.2 seconds per test image. In contrast, ResNet-50 and YOLOv2, using
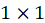
 images and having similar structures in their trained layers, deliver significantly faster results than the detection models designed for
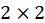
 images. Moreover, it's evident that larger input image sizes correspond to longer testing times. Notably, in the case of Faster R-CNN, when the input structure is halved in height, the testing time is also halved.

## Classification analysis

To quantitatively evaluate the influence of incorporating radiologist-defined ROI information and mosaic-based input structures on classification performance, an ablation study was conducted using ResNet-50 as the base network. Table S-1 summarizes the results for each configuration. While introducing ROI information alone (YOLOv2
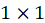
) did not improve overall accuracy compared to the baseline CNN, the combination of ROI guidance and spatially diversified mosaic inputs (YOLOv2
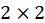
 and Faster R-CNN
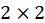
) yielded consistent improvements in all metrics. This finding suggests that ROI information must be integrated with sufficient contextual representation to achieve robust classification. Notably, Faster R-CNN
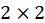
 achieved the highest sensitivity (0.8646), indicating superior detection of malignant nodules when both ROI and mosaic cues were available.

To further visualize classification outcomes, per-epoch confusion matrices were generated for each model, as shown in Figure S-2. These matrices illustrate epoch-wise convergence behavior and class-specific stability trends for benign and malignant nodules.

## ROI detection analysis

In this section, we compare the recognized ROI based on the heat map from originally trained CNN and the resulting ROI that is produced by newly trained CNN integrated with ROI learning. To examine how well the localization is performed, first, we consider images as malignant in each algorithm, meaning images where a true positive diagnosis of malignancy was obtained. First, we use ResNet-50 trained without ROI information to generate heat maps and select the top 50% or 70% of the heat map areas as ROI (referred to as region D in Section 3.1). Next, we adopt the regions proposed by the detection models (Faster R-CNN and YOLOv2) trained with ROI information as the ROI, derived from these models (also referred to as region D in Section 3.1).

To assess how well the ROIs derived from each algorithm align with the ROIs drawn by radiologists (referred to as region
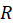
 in Section 3.1), we calculate the
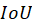
 between
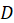
 and
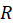
. We also observe what percentage of the total true positive (TP, malignant) images have
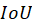
 values greater than a given threshold,
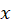
, as shown in Figure S-3. This ratio serves as an indicator of how much the algorithm-diagnosed images rely on the regions that should actually be examined when making a diagnosis. For instance, if the number in Table S-2 is 0.8276, it means that roughly 82.8% of the images were diagnosed as malignant based on feature analysis of the correct areas, while the remaining 17.2% were diagnosed as malignant based on data incorporating information from other regions. A representative example from this 17.2% subset is shown in Figure S-4 (a).

Table S-3 shows that even when analyzing benign images, they exhibit a similar pattern to malignant ones. Remarkably, standard ResNet-50 assigns a lower probability in finding proper ROI to benign images than to malignant ones. In the subset of true negative (TN, benign) images, we find that, even when we consider up to 70% of the heatmap values in the ROI as defined by the standard ResNet-50, only about 45% of these true negative images appear to base their decision on information from an ROI that aligns with the area identified by radiologists as worthy of observation. Essentially, any portions of the image that aren't indicative of malignant nodules are likely to be classified as benign. This means that, similar to the second image in Figure S-4 (b), ResNet-50 can classify benign images as such by examining areas beyond the ROI. However, ResNet-50 seems to face challenges when identifying negative nodules. In contrast, the results strongly support that over 94% of images correctly classified by both Faster R-CNN and YOLOv2, whether benign or malignant, are effectively identified by accurately pinpointing the ROI area.

## Results with Alternative CNN Backbones

The tests are conducted with GoogLeNet and Inception-v3. The detection and classification results of CNN combined with and without YOLOv2 for GoogLeNet and Inception-v3 are presented. To compare detection performance, CNN uses the top 70% of the score map created by Grad-CAM, and YOLOv2 combined with CNN uses the predicted bounding box. Table S-4 can be compared to Table III in the main text, and Table S-5 observes the same content as Table IV in the main text.

Additional References

1. M. T. GadAllah, A. E.-N. Mohamed, A. A. Hefnawy, et al., “Convolutional neural networks based classification of segmented breast ultrasound images – a comparative preliminary study,” in 2023 Intelligent Methods, Systems, and Applications (IMSA), 2023, pp. 585–590.
2. Y. Sharifi, M. A. Bakhshali, T. Dehghani, et al., “Deep learning on ultrasound images of thyroid nodules,” Biocybern. Biomed. Eng., vol. 41, pp. 636–655, 2021, doi: 10.1016/j.bbe.2021.02.008.
3. S. Gao, Y. Li, and H. Luo, “Detecting thyroid nodules along with surrounding tissues and tracking nodules using motion prior in ultrasound videos,” Computerized Medical Imaging and Graphics, vol. 117, p. 102439, 2024, doi: 10.1016/j.compmedimag.2024.102439.
4. Y. Liu, Y. Feng, L. Qian, Z. Wang, and X. Hu, "Deep learning diagnostic performance and visual insights in differentiating benign and malignant thyroid nodules on ultrasound images," Experimental Biology and Medicine, vol. 248, no. 24, pp. 2538–2546, Dec. 2023, Epub Jan. 26, 2024, doi: 10.1177/15353702231220664.
5. A. Radhachran, A. Kinzel, J. Chen, V. Sant, M. Patel, R. Masamed, C. W. Arnold, and W. Speier, "A multitask approach for automated detection and segmentation of thyroid nodules in ultrasound images," Computers in Biology and Medicine, vol. 170, p. 107974, 2024, doi: 10.1016/j.compbiomed.2024.107974.
6. X. Yang, H. Geng, X. Wang, G. Hu, Y. Li, P. Ma, H. Cheng, W. Chen, Z. Wen, Q. Li, and J. Wang, "Identification of lesion location and discrimination between benign and malignant findings in thyroid ultrasound imaging," Scientific Reports, vol. 14, no. 1, p. 32118, 2024, doi: 10.1038/s41598-024-83888-1.
7. J. Wang, X. Yang, X. Jia, W. Xue, R. Chen, Y. Chen, X. Zhu, L. Liu, Y. Cao, J. Zhou, D. Ni, and N. Gu, "Thyroid ultrasound diagnosis improvement via multi-view self-supervised learning and two-stage pre-training," Computers in Biology and Medicine, vol. 171, p. 108087, 2024, doi: 10.1016/j.compbiomed.2024.108087.
8. G. Chen, G. Tan, M. Duan, B. Pu, H. Luo, S. Li, and K. Li, "MLMSeg: A multi-view learning model for ultrasound thyroid nodule segmentation," Computers in Biology and Medicine, vol. 169, p. 107898, 2024, doi: 10.1016/j.compbiomed.2023.107898.
9. Y. Zhou, C. Chen, J. Yao, et al., "A deep learning based ultrasound diagnostic tool driven by 3D visualization of thyroid nodules," npj Digital Medicine, vol. 8, p. 126, 2025, doi: 10.1038/s41746-025-01455-y.
10. J. Yang, Z. Luo, Y. Wen, and J. Zhang, "Artificial intelligence-enhanced ultrasound imaging for thyroid nodule detection and malignancy classification: a study on YOLOv11," Quantitative Imaging in Medicine and Surgery, vol. 15, no. 9, pp. 7964-7976, 2025, doi: 10.21037/qims-2025-257.
11. G. Fu, G. Gu, W. Liu, and H. Fu, "LISA-YOLO: A symmetry-guided lightweight small object detection framework for thyroid ultrasound images," Symmetry, vol. 17, no. 8, p. 1249, Aug. 2025, doi: 10.3390/sym17081249.
12. Y. Xu, M. Xu, Z. Geng, J. Liu, and B. Meng, "Thyroid nodule classification in ultrasound imaging using deep transfer learning," BMC Cancer, vol. 25, no. 1, p. 544, 2025, doi: 10.1186/s12885-025-13917-3.
13. Y. Xiang, R. Acharya, Q. Le, J. Tan, and C.-L. Chng, "Thyroid nodule segmentation in ultrasound images using transformer models with masked autoencoder pre-training," Frontiers in Artificial Intelligence, vol. 8, p. 1618426, 2025, doi: 10.3389/frai.2025.1618426.
14. Y. Sharifi, M. D. Ashgari, S. Shafiei, S. R. Zakavi, and S. Eslami, "Using deep learning for thyroid nodule risk stratification from ultrasound images," WFUMB Ultrasound Open, vol. 3, no. 1, p. 100082, 2025, doi: 10.1016/j.wfumbo.2025.100082.
15. S. Wang, Z.-A. Zhao, Y. Chen, Y.-J. Mao, and J. C.-W. Cheung, “Enhancing thyroid nodule detection in ultrasound images: A novel YOLOv8 architecture with a C2fA module and optimized loss functions,” Technologies, vol. 13, no. 1, p. 28, 2025, doi: 10.3390/technologies13010028.
16. X. Wang, Y. Niu, H. Liu, F. Tian, Q. Zhang, Y. Wang, Y. Wang, Y. Li, "ThyroNet-X4 genesis: An advanced deep learning model for auxiliary diagnosis of thyroid nodules’ malignancy," Scientific Reports, vol. 15, no. 1, p. 4214, 2025, doi: 10.1038/s41598-025-86819-w.
17. X. Cai, Y. Zhou, J. Ren, J. Wei, S. Lu, H. Gu, W. Xu, and X. Zhu, "Intelligent diagnosis of thyroid nodules with AI ultrasound assistance and cytology classification," Frontiers in Endocrinology, vol. 16, p. 1546983, 2025, doi: 10.3389/fendo.2025.1546983.
18. H. Bi, C. Cai, J. Sun, S. Ge, H. Shu, and X. Ni, “DRTNet: Dual-route transformer network for thyroid ultrasound segmentation based on Bbox-supervised learning,” Knowledge-Based Systems, vol. 324, Art. no. 113781, 2025, doi: 10.1016/j.knosys.2025.113781.
19. J. Liu, Y. Peng, M. Pu, L. Tang, L. Shao, P. Wang, L. Yang, F. Huang, and Z. Shen, "KTUNet: A hybrid CNN-Transformer with Kolmogorov-Arnold convolution for thyroid nodule segmentation in ultrasound images," Digital Medicine, vol. 11, no. 2, pp. e24-00019, Jun. 2025, doi: 10.1097/DM-2024-00019.
20. N. Wang, R. Zhang, L. Dong, G. Zhang, and S. Meng, "Improving the diagnosis of endometrial cancer in postmenopausal women in primary care settings using an artificial intelligence-based ultrasound detecting model," Frontiers in Oncology, vol. 15, p. 1646826, 2025, doi: 10.3389/fonc.2025.1646826.
21. J. Kim, M. H. Kim, D. J. Lim, H. Lee, J. J. Lee, H. S. Kwon, M. K. Kim, K. H. Song, T. J. Kim, S. L. Jung, Y. O. Lee, and K. H. Baek, "Deep learning technology for classification of thyroid nodules using multi-view ultrasound images: potential benefits and challenges in clinical application," Endocrinology and Metabolism, vol. 40, no. 2, pp. 216–224, 2025, doi.org/10.3803/EnM.2024.2058.

**Figure S-1.** Training and validation curves for representative models: Each curve illustrates convergence stability and agreement between training and validation metrics (a) ResNet-50 (1×1) – training and validation accuracy/loss

(b) YOLOv2 (2×2) – training and validation RMSE/loss (c) Faster R-CNN (1×2) – training and validation accuracy/loss.

| (a) | 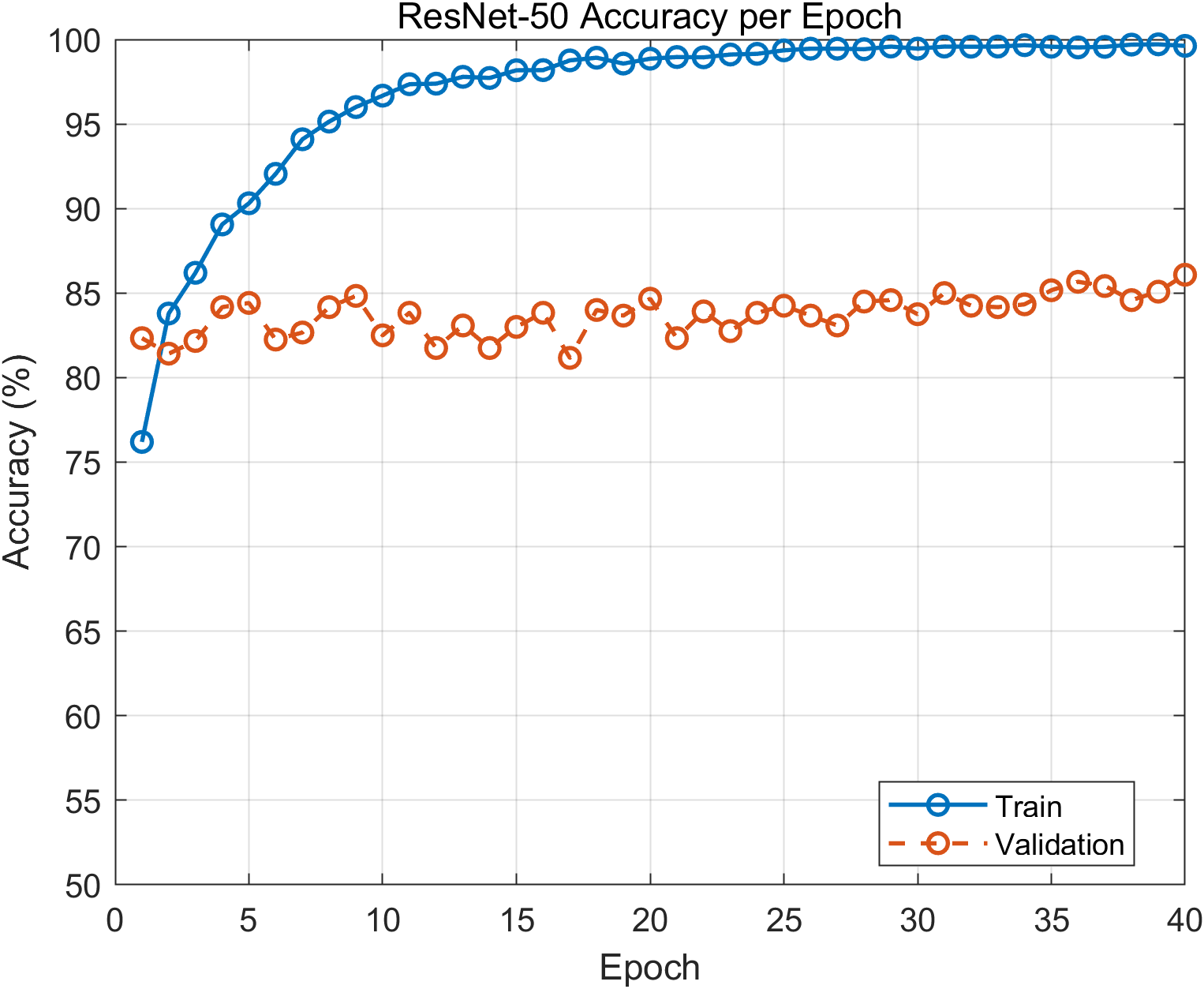 | 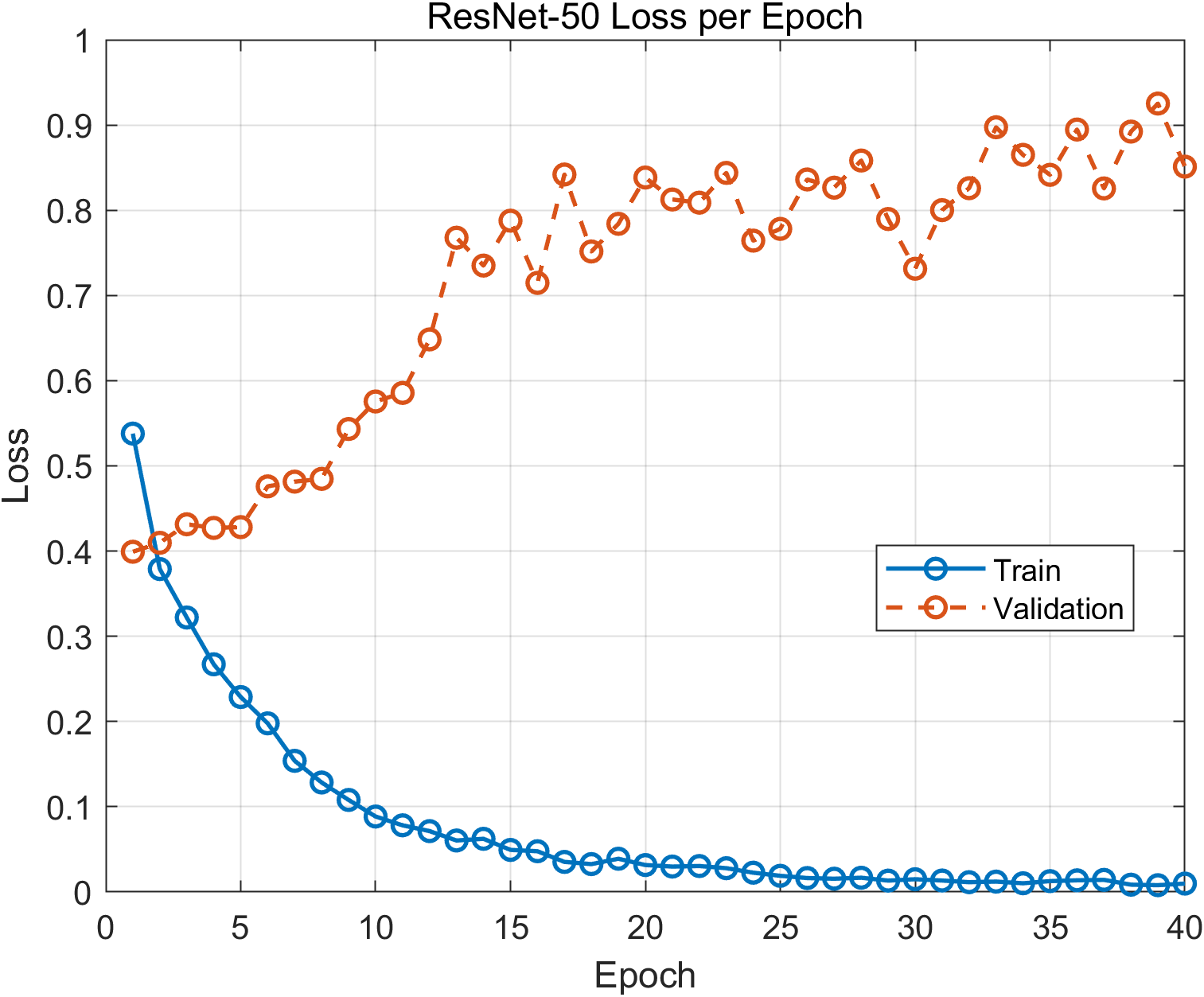 |
| --- | --- | --- |
| (b) | 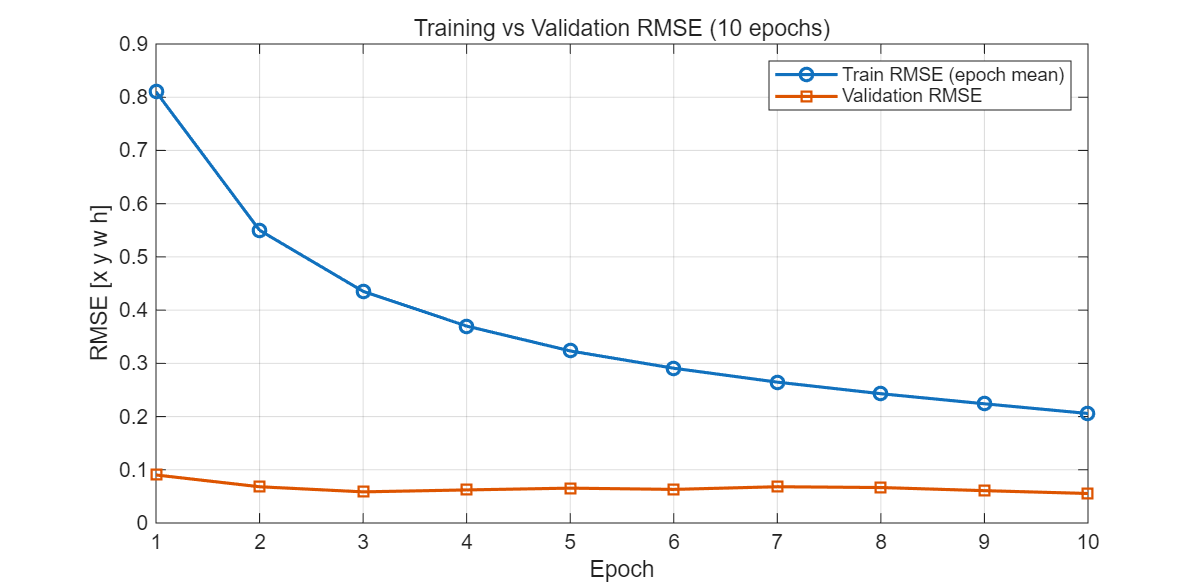 | 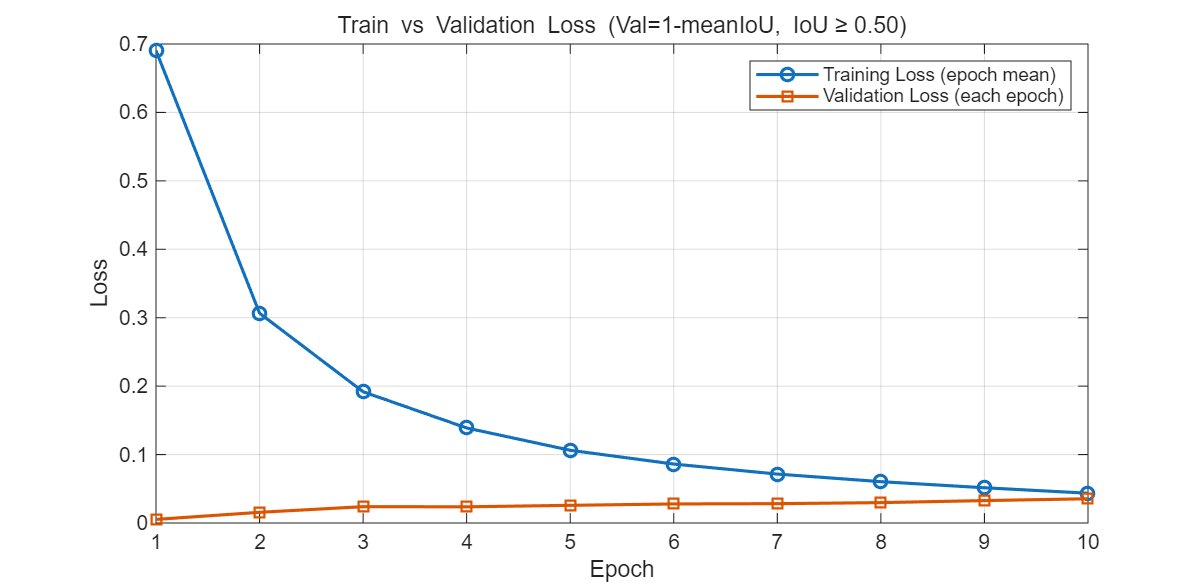 |
| (c) | 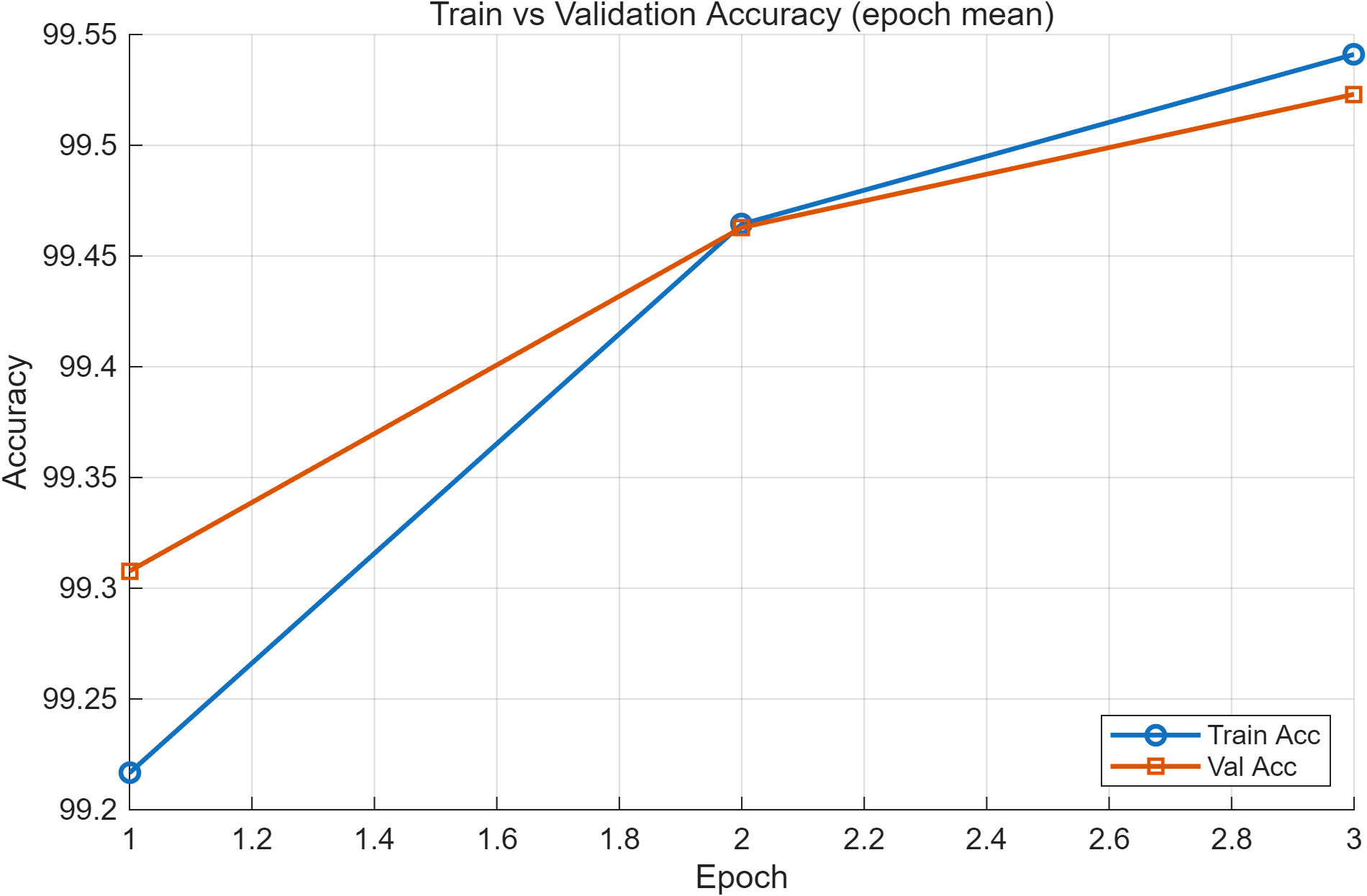 | 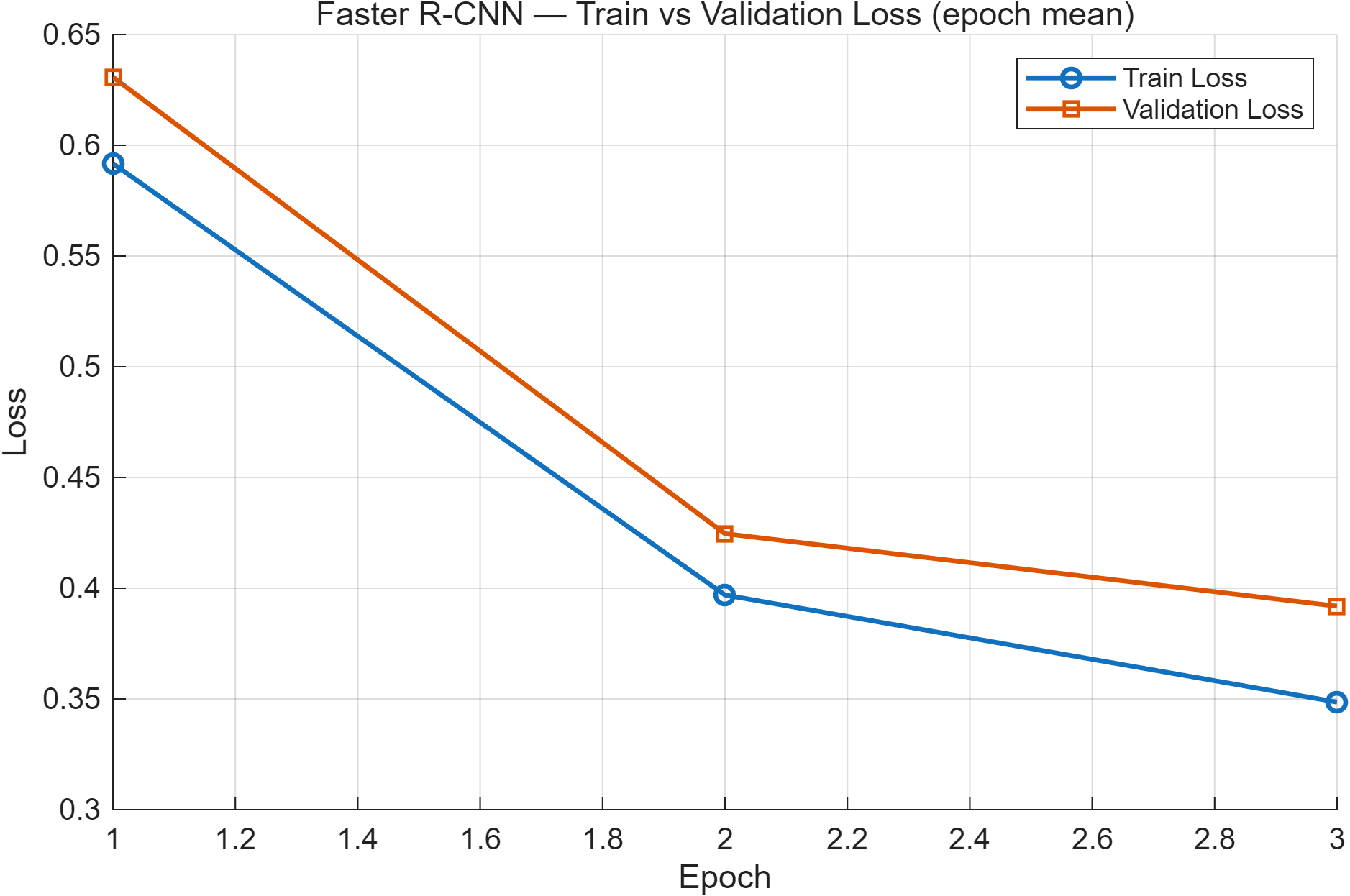 |

(a)
(b)

(c)

(d)
(e)

**Figure S-2.**

Confusion matrices illustrating classification outcomes for each model configuration. Each confusion matrix displays classification results for benign and malignant nodules, where the left-side row labels (“Act: Pos”, “Act: Neg”) denote the actual ground-truth classes, and the bottom column labels (“Pred: Pos”, “Pred: Neg”) represent the predicted classes. True positive (TP), false positive (FP), true negative (TN), and false negative (FN) counts are shown numerically within each grid cell. Subfigures (a)–(c) depict epoch-wise stability over epochs 31–40, whereas (d)–(e) show final epoch results (epoch 3) for Faster R-CNN.

**Figure S-3.** Graphs depicting the change in probability values for each
. The probability is the ratio of images satisfies that
 among malignant images classified as malignant by each model.


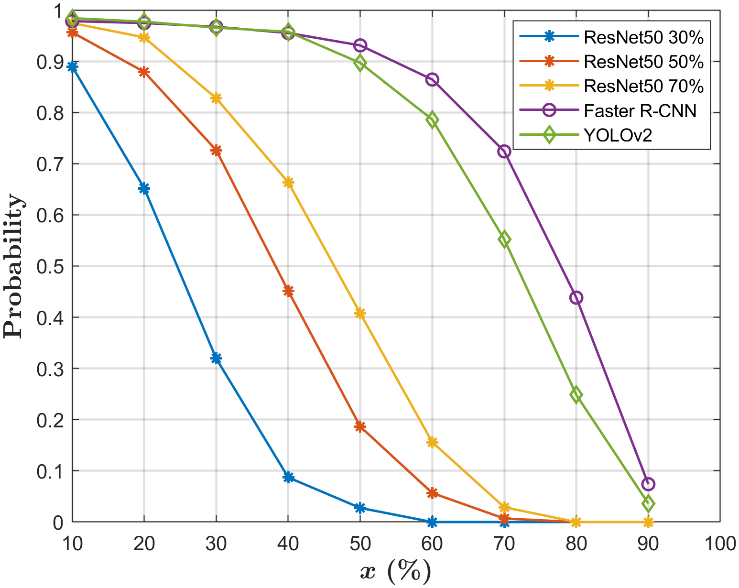


(a) (b) (c) (d)

**Figure S-4.** The original images with the ROI and detection areas of ResNet-50, Faster R-CNN, and YOLOv2 from the left. (a) a malignant image classified as malignant by all three models. (b) a benign image classified as benign by all three models. (c) a malignant image classified as malignant by Faster R-CNN and YOLOv2, and as benign by ResNet-50. (d) a benign image classified as benign by Faster R-CNN and YOLOv2, and as malignant by ResNet-50.

TABLE S-1

Ablation study on the effect of ROI integration and mosaic structure: The table compares models with and without radiologist-defined ROI guidance and mosaic input structures. Values for ResNet-50 and YOLOv2 models represent the mean over epochs 31–40, while Faster R-CNN values correspond to the final (3rd) epoch due to longer training time.

| Model | Epoch | ROI | Mosaic | Accuracy | Specificity | Sensitivity |
| --- | --- | --- | --- | --- | --- | --- |
| ResNet-50 (1×1) | 31-40 | X | X | 0.8038 | 0.7726 | 0.8093 |
| YOLOv2 + ResNet-50 (1×1) |  | O | X | 0.7087 | 0.8653 | 0.6811 |
| YOLOv2 + ResNet-50 (2×2) |  | O | O | 0.8446 | 0.8000 | 0.8525 |
| Faster R-CNN + ResNet-50 (1×2) | 3 | O | O | 0.8375 | 0.7684 | 0.8497 |
| Faster R-CNN + ResNet-50 (2×2) |  | O | O | 0.8549 | 0.8000 | 0.8646 |

TABLE S-5

Comparison of classification results of CNN combined with and without YOLOv2 for GoogLeNet and Inception-v3.

| Model | GoogLeNet | GoogLeNet with  YOLOv2 | Inception-v3 | Inception-v3 with  YOLOv2 |
| --- | --- | --- | --- | --- |
| Input structure | 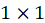 | 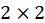 | 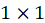 | 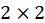 |
| Accuracy | 0.8233 | 0.8443 | 0.7533 | 0.8358 |
| Specificity | 0.8463 | 0.7642 | 0.8358 | 0.6379 |
| Sensitivity | 0.8193 | 0.8584 | 0.7388 | 0.8707 |

TABLE S-2

The probability is the ratio of images satisfies that IoU≥30 % among TP images.

| Model | | 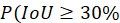 | TP, 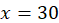) |
| --- | --- | --- |
| ResNet-50 | ROI determined by top 30% of heatmap value | 0.3193 |
|  | ROI determined by top 50% of heatmap value | 0.7263 |
|  | ROI determined by top 70% of heatmap value | 0.8276 |
| ResNet-50 with Faster R-CNN | | 0.9656 |
| ResNet-50 with YOLOv2 | | 0.9673 |

TABLE S-3

The probability is the ratio of images satisfies that IoU≥30 % among TN images.

| Model | | 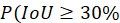 | TN, 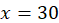) |
| --- | --- | --- |
| ResNet-50 | ROI determined by top 30% of heatmap value | 0.1144 |
|  | ROI determined by top 50% of heatmap value | 0.2452 |
|  | ROI determined by top 70% of heatmap value | 0.4578 |
| ResNet-50 with Faster R-CNN | | 0.9452 |
| ResNet-50 with YOLOv2 | | 0.9598 |

TABLE S-4

Comparison of detection results of CNN combined with and without YOLOv2 for GoogLeNet and Inception-v3.

| Metric | GoogLeNet | | Inception-v3 | |
| --- | --- | --- | --- | --- |
|  | Top 70% of heatmap | YOLOv2 | Top 70% of heatmap | YOLOv2 |
| 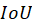 | 15.43% | 72.26% | 37.99% | 72.15% |
| 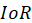 | 39.67% | 82.08% | 63.03% | 85.54% |
| 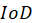 | 24.73% | 88.26% | 52.46% | 84.45% |

TABLE S-6

Overview of recent deep learning studies (2024–2025) on thyroid ultrasound imaging, summarizing task type, ROI usage, model architecture, evaluation metrics, dataset details, and validation scope.

| **Year** | **Study** | **Task** | **Input** | **ROI use** | **Backbone/Detector** | **Key metric(s)** | **Data size** | **External val.** | **Notes** |
| --- | --- | --- | --- | --- | --- | --- | --- | --- | --- |
| 2024 | Vahdati et al. [16] | Detection + Classification | Multi-view (transverse + longitudinal) | Explicit (bbox) | YOLOv5 ×2 + XGBoost | AUROC 0.84; Sensitivity 84%; Specificity 63%; mAP@0.5 0.797 / 0.716 | 983 patients; 5-fold CV; 81 held-out test; single center | No | Two-view ensemble; NMS (Non-Maximum Suppression),XGBoost fusion; held-out internal test only |
| 2024 | Zhou et al. [17] | Detection | 1×1 | Explicit | DN-DETR with Deformable Conv v2, MHSA, BatchFormerV2 | mAP@0.5: 0.909, AP: 0.529, AUC: 0.826; Improvement over DN-DETR baseline (mAP +0.083) | 8201 ultrasound images (6800 train, 701 val, 700 test); single hospital dataset | Not reported | Transformer-based DETR model optimized with deformable convolution v2; multi-head self-attention, and dual-stream BatchFormerV2; uses transfer learning and GELU activation |
| 2024 | Gao et al. [22] | Detection (+Tracking) | Video (thyroid ultrasound videos, >30 frames) | Explicit | Custom detection pipeline DiffusionVID-Line + tracking ByteTrack-Line | mAP50: 74.2 (multiple tissues), 85.6 (nodules); MOTA (tracking): 83.4 | Patient ultrasound videos (single hospital dataset) | Not reported | Leverages linear motion prior; frequency feature extraction (Freq-FPN); attention on linear motion (Attn-Line); improves detection & tracking accuracy |
| 2024 | Liu et al. [23] | Classification | 1×1 | Weak (Grad-CAM) | ResNet series | Accuracy 73%; Sensitivity 81.67%; Specificity 60%; AUC 0.79 | 2096 US images, 655 nodules (single center) | No | AI vs 80 radiologists; Grad-CAM for visual explanation; Turing test |
| 2024 | Radhachandran et al. [24] | Detection + Segmentation | 1×1 | Explicit | Multitask CNN with anomaly detection + U-Net | F1: 0.839 Dice: 0.808 | 9888 US images from 280 patients (single center) | Yes | Multitask anomaly detection and segmentation; externally validated |
| 2024 | Yang et al. [25] | Detection + Classification | 1×1 | Explicit | YOLOv8-L | Detection: mAP@50 = 95.5%; Classification Precision: 88.5% (benign), 89.3% (malignant); Recall: 89.3% (benign), 90.4% (malignant) | Single institution, 3055 US images of 3088 nodules (benign: 1752, malignant: 1336) +DDTI test | Yes | Attention-weighted DCN, CPCA (Channel prior convolutional attention), feature fusion network, transfer learning |
| 2024 | Wang et al. [26] | Classification + Segmentation | Multi-view US | Mixed (weak + explicit masks + visualization) | CNN encoder + Contrastive self-supervised learning | Classification AUC: up to 0.86 (multi-view), Dice: up to 0.87 | 9669 US images, 5224 patients, multi-center (20+ hospitals) | No | Multi-view self-supervised; robust to missing views; two-stage pretraining |
| 2024 | Chen et al. [27] | Segmentation | Multi-view | Mixed (Weak + Explicit mask + Visualization) | CNN encoder + multi-channel Transformer + Graph Convolution Network (MLMSeg) | Dice: 92.10% (DTN), 83.84% (DDTI); IoU: 86.60% (DTN), 73.52% (DDTI) | DTN, DDTI, DCR, DHF datasets (multi-center) | Yes | MLMSeg; multi-view fusion; strong cross-dataset results |
| 2025 | Zhou et al. [28] | Classification (+ video assist) | Video / 3D visualization | Weak (visualization focused) | Two-stage deep learning framework with YOLOv8 and Swin-Unet | Internal Dice Similarity Coefficient (DSC) 0.90; external AUC 0.79 (significant improvement of radiologists) | Development: 4,569 cases; Validation: 7 hospitals; multi-center | Yes | Dynamic ultrasound; 3D visualization aid |
| 2025 | Yang et al. [29] | Detection + Classification | 1×1 (with video capability) | Explicit | YOLOv11n | Precision: 0.841, Recall: 0.823 @IoU 0.5 | 1,503 US images (742 benign, 778 malignant) from Roboflow open dataset | No | Real-time AI tool for ultrasound; lightweight and fast YOLOv11 variant validated vs YOLOv3–10 and ResNet50 |
| 2025 | Fu et al. [30] | Detection | 1×1 | Explicit | Lightweight YOLO variant with DG-FNET, IMSF-NET, SAF-NET modules | mAP@50: 83.4%; F1 ↑2.3; FPS ↑9.0; Params: 2.6M; GFLOPs: 5.8 | Self-built + Public thyroid US datasets | Not reported | Small-lesion focus; symmetry multi-scale fusion; collab attention; faster than YOLOv11; COCO pretrained weights not used |

TABLE S-6 (continued)

| **Year** | **Study** | **Task** | **Input** | **ROI use** | **Backbone/Detector** | **Key metric(s)** | **Data size** | **External val.** | **Notes** |
| --- | --- | --- | --- | --- | --- | --- | --- | --- | --- |
| 2025 | Xu et al. [31] | Classification | 1×1 | None | InceptionV3 (Transfer Learning) + SVM fusion | SVM AUC 0.748 (95% CI 0.684–0.811); Inception V3 AUC 0.763 (95% CI 0.702–0.825); Fusion AUC 0.783 (95% CI 0.724–0.841, p = 0.036); Accuracy 72.2% (Inception V3) | 1134 US images from 630 cases (589 benign, 545 malignant) | No | LASSO feature selection; SVM and Inception V3 post-fusion model; Delong test; DCA clinical utility; retrospective single-center study |
| 2025 | Xiang et al. [32] | Segmentation | 1×1 | Explicit (mask) | Transformer-based model with Masked Autoencoder pre-training | IoU and Dice scores: DSC 0.63-0.65 | Small-sample multi-center public datasets (AIMI, TN3K, DDTI) | Not reported | Transformer global context; MAE pretrain; data volume limits accuracy; cross-attention study |
| 2025 | Sharifi et al. [33] | Classification (Risk stratification) | 1×1 ultrasound images | Explicit (ACR-TIRADS) | Faster R-CNN with ResNet-101 (detection), Xception (classification) | Accuracy: 0.98; AUC: 0.99; Precision: 0.967; Recall: 0.912 | 2450 US images, 3250 nodules, 1037 patients, single center | No | DL based CAD on ACR-TIRADS; outperformed experts; large curated dataset |
| 2025 | Wang et al. [34] | Detection | 1×1 | Explicit | YOLOv8 + C2fA (CoordAtt) + CW-BCE + SIoU | Precision: 0.54, Recall: 0.582 | Public DDTI dataset (339 images) | No | Lightweight YOLOv8 with CW-BCE and SIoU; improved localization and balance; detects malignant-feature nodules |
| 2025 | Wang et al. [35] | Classification | 1×1 | None | ResNet-based ThyroNet-X4 (CNN) | Accuracy: 71.7% (validation), 67.0% (external) | Public datasets + external hospital dataset | Yes | Multi-module CNN; good generalization via external test |
| 2025 | Cai et al. [36] | Classification (Multimodal) | Ultrasound image + Clinical + Cytology | Explicit | AI Ultrasound-assisted system + ML (Random Forest best) | AUC: 0.994 (training), 0.993 (testing), 0.977 (prospective); Accuracy: ~90.48% for ITNs | 620 + prospective 243 + 70; single center | Yes | Multimodal fusion (AI-US + FNAC + demo); optimized for indeterminate nodules |
| 2025 | Bi et al. [37] | Segmentation | 1×1 | Explicit | Dual-route Transformer (DRTNet) | Dice: 84.94% (TN3k), 83.98% (DDTI); HD95: 27.69 (TN3k), 29.18 (DDTI) | TN3k, DDTI + private set | Not reported | Bbox-supervised transformer; dual-branch CAMs + uncertainty regularization |
| 2025 | Liu et al. [38] | Segmentation | 1×1 | Explicit | Hybrid CNN + Transformer (KTUNet) | Dice 82.5 %(TN3K), ≈ 85 %(private);  IoU ≈ 80 % | TN3K + private set | Not reported | Hybrid KAC–CNN–Transformer; enhanced nonlinear and global feature fusion |
| 2025 | Shen et al. [18] | Classification (Multimodal, Explainable AI) | 1×1 ultrasound + clinical text + demographics | Explicit (YOLOv8 + U-Net++ segmentation) | YOLOv8 + U-Net++ + Transformer fusion (LLNM-Net) | AUC: 0.944 (multi-center); Accuracy: 84.7% | 29 615 patients across 7 centers (9836 surgical cases) | Yes | Multimodal XAI; bidirectional attention fusion; outperforms experts |
| 2025 | Lin et al., [19] | Detection + Classification (video screening) | Cranial US videos → multi-view | Explicit (auto standard-view extraction) | NCLS two-stage pipeline (view extractor + multi-view classifier) | AUC 0.982 (internal), 0.944 (external); Sensitivity 0.875 / 0.962; Specificity 0.934 / 0.927 | Dev: 8,757 images, 1,518 cases (single center); Test: 199 internal cases; 356 external cases  (3 centers) | Yes | Extracts standard views; faster than juniors; assists diagnosis of severe lesions |
| 2025 | Wang et al. [39] | Detection  (+ binary classification of EC) | 1×1 grayscale US + Doppler | Explicit (Bounding Box) | YOLOv8-S with deformable conv  + dynamic prototype loss | AUC: 0.844 (train), 0.811 (val), 0.858 (test); AP@0.5: 0.82; Acc 85.8%, Sens 77.3%, Spec 90.1% (test) | 877 patients, single center; split 614/175/88; 7,544 train images | No | Mosaic augmentation; Grad-CAM; compares vs endometrial thickness  (AUC 0.734) |
| 2025 | Kim et al. [40] | Classification | 1×1 (multi-view US) | None / Weak (with visualization) | Deep Learning classifiers  (ResNet, DenseNet, EfficientNet) | Accuracy 0.81~0.83; Specificity 0.79~0.92; AUC ~0.8-0.88 | 1,048 nodules from 943 patients (single center) | No | Paired transverse/longitudinal; CNN architectures compared; improved accuracy with higher resolution images |

1.  [↑](#footnote-ref-2)
